# Development of an ensemble CNN model with explainable AI for the classification of gastrointestinal cancer

**DOI:** 10.1371/journal.pone.0305628

**Published:** 2024-06-25

**Authors:** Muhammad Muzzammil Auzine, Maleika Heenaye-Mamode Khan, Sunilduth Baichoo, Nuzhah Gooda Sahib, Preeti Bissoonauth-Daiboo, Xiaohong Gao, Zaid Heetun

**Affiliations:** 1 Department of Software and Information Systems, University of Mauritius, Reduit, Mauritius; 2 Department of Computer Science, Middlesex University London, London, United Kingdom; 3 Center for Gastroenterology and Hepatology, Dr Abdool Gaffoor Jeetoo Hospital, Port Louis, Mauritius; University of Manitoba, CANADA

## Abstract

The implementation of AI assisted cancer detection systems in clinical environments has faced numerous hurdles, mainly because of the restricted explainability of their elemental mechanisms, even though such detection systems have proven to be highly effective. Medical practitioners are skeptical about adopting AI assisted diagnoses as due to the latter’s inability to be transparent about decision making processes. In this respect, explainable artificial intelligence (XAI) has emerged to provide explanations for model predictions, thereby overcoming the computational black box problem associated with AI systems. In this particular research, the focal point has been the exploration of the Shapley additive explanations (SHAP) and local interpretable model-agnostic explanations (LIME) approaches which enable model prediction explanations. This study used an ensemble model consisting of three convolutional neural networks(CNN): InceptionV3, InceptionResNetV2 and VGG16, which was based on averaging techniques and by combining their respective predictions. These models were trained on the Kvasir dataset, which consists of pathological findings related to gastrointestinal cancer. An accuracy of 96.89% and F1-scores of 96.877% were attained by our ensemble model. Following the training of the ensemble model, we employed SHAP and LIME to analyze images from the three classes, aiming to provide explanations regarding the deterministic features influencing the model’s predictions. The results obtained from this analysis demonstrated a positive and encouraging advancement in the exploration of XAI approaches, specifically in the context of gastrointestinal cancer detection within the healthcare domain.

## 1 Introduction

**Fig 1:**
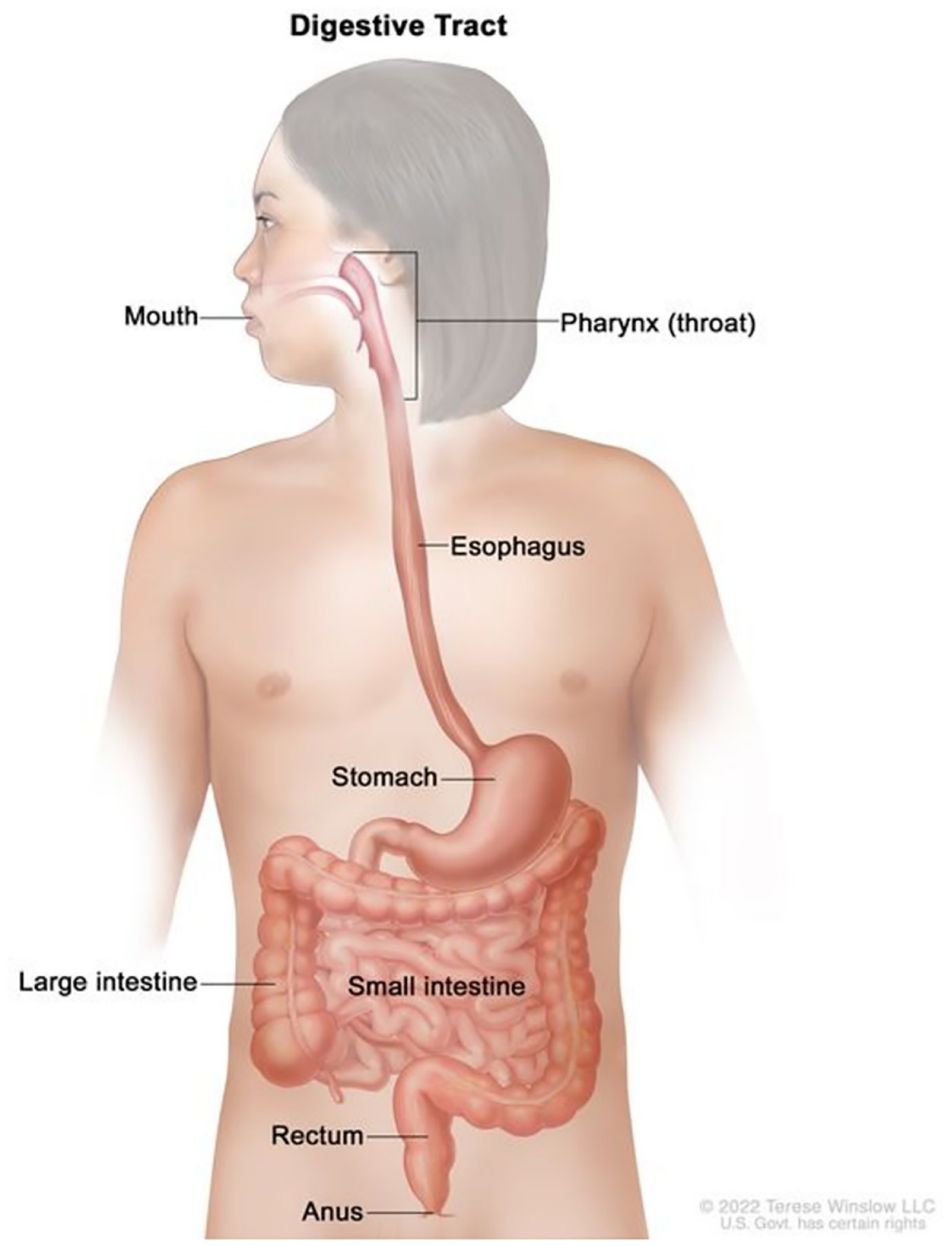
GI tract [4].

The gastrointestinal tract comprises of organs that form the digestive system. Mutation of cells lining at least one of these organs induces the production of tumors which eventually leads to the development of gastrointestinal cancer. It is noteworthy that gastrointestinal (GI) cancers have a significant global impact, accounting for approximately 26.3% of all cancer incidence cases (4.8 million cases) and 35.4% of cancer related deaths (3.4 million deaths) [1]. The gastrointestinal tract, as depicted in Fig 1, encompasses a lengthy pathway spanning approximately 25 feet, starting from the mouth and ending at the anus. Several studies, including [2, 3], have identified the most prevalent types of gastrointestinal cancers, which include gastric (stomach) cancer, esophageal cancer, colorectal cancer, Pancreatic cancer and liver cancer.

Latest studies have reported that a substantial proportion (over 50%) of gastrointestinal cancers can be attributed to risk factors that can be altered by adopting a healthier lifestyle, alcohol intake, cigarette smoking, infection, unhealthy diet and obesity [5, 6]. Moreover, it has been observed that males have a higher susceptibility to gastrointestinal cancers compared to females, with the risk increasing with age, as indicated by [2]. Unfortunately, due to late stage diagnoses being predominant, the prognosis for such cancers is typically unfavorable [7], thus resulting in site specific death rates that align with the incidence trends. However, if gastrointestinal cancers are detected in their early stages, the survival rate becomes higher in the five year timeline [8]. Nonetheless, a study conducted by [9] put forward that cognitive and technological issues contribute to significant diagnostic errors, despite the effectiveness of traditional screening procedures.

The Global Cancer Observatory [1] predicts a substantial increase in the global mortality and incidence rates of GI cancers by the year 2040. The mortality rate is projected to rise by 73%, reaching approximately 5.6 million cases, while the incidence rate is expected to increase by 58%, with an estimated 7.5 million new cases. These alarming statistics highlight the urgent need for the development of dependable systems to support medical facilities in obtaining accurate GI cancer diagnoses. Addressing this priority through innovative research endeavors becomes crucial in order to effectively combat the rising burden of GI cancers on a global scale.

Recent research has highlighted the potential of artificial intelligence (AI) in reducing misdiagnosis rates associated with conventional screening techniques, thereby enhancing overall diagnostic accuracy [10]. This achievement is primarily attributed to the utilisation of deep learning and machine learning algorithms. However, a significant hurdle faced by AI supported systems is their perceived nature as computational “black boxes”. The lack of transparency in the decision making processes of these AI models has resulted in hesitancy among healthcare institutions when it comes to adopting them for diagnostic purposes, despite their effectiveness [11, 12]. It is therefore important for AI researchers to integrate digestible explanations throughout the development of AI aided medical applications, thus assuring healthcare practitioners while also clearing any doubts they might have. In this context, explainable artificial intelligence (XAI) has emerged as a promising field that aims to address the computational difficulties posed by AI systems, warranting the provision of explanations for model predictions [13]. By employing f techniques, AI researchers can enhance the interpretability and transparency of AI driven diagnostic systems, thereby fostering trust and facilitating their integration into clinical practice.

To address the aforementioned challenges in AI driven diagnostic systems, this research work focuses on the investigation of Shapley additive explanations (SHAP), introduced by [14] and local interpretable model-agnostic explanations (LIME), introduced by [15]. In our study, we have utilised an ensemble model that we developed and trained on the pathology results obtained from the publicly accessible Kvasir dataset. By employing SHAP and LIME, we aim to provide interpretable explanations for the predictions made by our ensemble model, thereby enhancing the transparency and understandability of the AI assisted diagnostic.

This research paper introduces a novel methodology for the classification of gastrointestinal lesions, aiming to identify the crucial factors that impact the decision making process. The paper is structured as follows:

- Section 2 provides an overview of the current advancements in the classification of gastrointestinal cancers, offering insights into the existing research in this field through a comprehensive background study.
- Section 3 delves into the system architecture and outlines the specific models employed in this study for the classification of gastrointestinal lesions. The methodology and technical details of the models are described in this section.
- In Section 4, the results obtained from the classification process are presented and meticulously analysed. This section includes a thorough examination of the performance metrics and an evaluation of the model’s effectiveness in accurately classifying gastrointestinal lesions.
- Finally, Section 5 concludes the research paper by summarising the main findings and implications derived from the study. This section highlights the magnitude of the research in fostering the branch of gastrointestinal lesion classification and discusses potential future directions for further investigation.

The research paper makes several notable contributions, which are outlined as follows:

1. A novel enhanced XAI-based ensemble model has been developed upon the architectures of InceptionV3, InceptionResNetV2, and VGG16.
2. Our XAI-based ensemble model has surpassed the performance of current GI lesions classification techniques.
3. Our XAI-based ensemble model can determined and highlight the deterministic features of esophagitis, polyps and ulcerative colistis

## 2 Literature review

**Fig 2:**
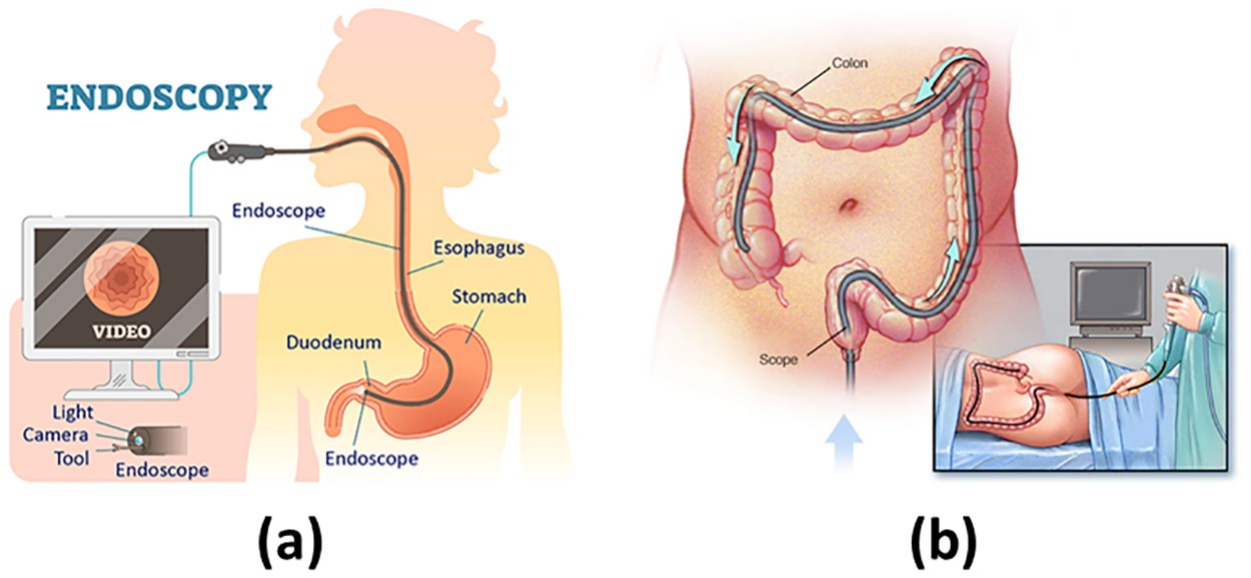
(a): Endoscopy procedure. [17], (b): Colonoscopy procedure. [18].

Endoscopy, in comparison to computed tomography (CT) scan and magnetic resonance imaging (MRI), is considered as the most effective method of screening for gastrointestinal cancer. This is primarily due to its ability to comprehensively examine the entire gastrointestinal tract and perform interventions such as the excision of polyps in a singular appointment if needed [16]. Abnormalities in the upper gastrointestinal tract, comprising of the stomach and oesophagus, are generally identified through endoscopy. The procedure involves the insertion of an endoscope through the mouth which spans the length of the oesophagus and ends at the duodenum, as depicted in Fig 2a. An endoscope constitutes of a camera, source of light and a tool channel. By utilising endoscopy, medical professionals can visually inspect the upper gastrointestinal tract and obtain valuable diagnostic information.

When considering the screening of the lower gastrointestinal tract, which includes the anus, rectum, and cecum, colonoscopy emerges as the favored approach. The procedure entails the insertion of a colonoscope, a subtype of endoscope, through the anus. With careful guidance, the colonoscope navigates the length of the colon until it reaches the cecum, as represented in Fig 2b. This process allows to visually examine the lower gastrointestinal tract, facilitating the identification of abnormalities such as polyps or lesions. Furthermore, if necessary, the colonoscopy procedure permits the collection of tissue samples to facilitate subsequent analysis. Given its capacity for direct visualisation and potential intervention, colonoscopy is an essential screening tool in the early detection and prevention of colorectal cancer.

Numerous research investigations have been carried out to develop automated models for detecting gastrointestinal cancer. According to [19], the detection of esophageal cancer using deep learning and machine learning is becoming progressively prevalent. Preliminary screening of esophageal cancer has been made possible through the development of computer assisted application by [20]. Eventually, the researchers achieved the classification of esophageal images through the implementation of random forest as an ensemble classifier for the classification of esophageal images. Nonetheless, deep learning models are being investigated.

In a study conducted in 2019, [21] developed a VGG16, InceptionV3, and ResNet50 model based on the transfer learning approach to classify endoscopic images into three classes: normal, benign ulcer, and cancer using a custom dataset of 787 images including 367 samples of cancer, 200 samples of normal cases, and 220 samples of ulcers collected from Gil Hospital. The images were first resized to 224 × 224 before they were preprocessed using adaptive histogram equalisation (AHE) to eliminate variations in the image brightness and contrast, thereby improving the local contrast and enhancing edge definition within each image region. Three binary classification tasks namely: normal vs. cancer, normal vs. ulcer, and cancer vs. ulcer were performed in this study and the accuracy, standard deviation, and area under the curve (AUC) values across the different convolutional neural networks(CNN) models. ResNet50 demonstrated the highest performance for all three performance metrics. The model achieved an accuracy of above 92% for the classification tasks including the normal images. However, for the cancer vs. ulcer task, a lower accuracy of 77.1% were noted. The authors conclude that this decrease is probably attributed due to the smaller visual differences between cancer and ulcer instances. ResNet50 also achieved the lowest standard deviation, which indicates greater stability among the other models. In terms of AUC, ResNet50 reported an AUC of 0.97, 0.95, and 0.85, respectively for the normal vs. ulcer, normal vs. cancer, and cancer vs. ulcer tasks. The authors concluded that this proposed deep learning approach can be a valuable tool to complement traditional screening practices by medical practitioners thus reducing the risk of missing positive cases due to repetitive endoscopic frames or diminishing concentration.

[22] developed a deep CNN based on the UNet++ and Resnet50 architectures to classify between cases of gastritis (AG) and non-atrophic gastritis (non-AG) using white light endoscopy images. A total of 6,122 images (4,022 AG cases and 2,100 non-AG) were collected from 456 patients and were randomly partitioned into training (89%) and test sets (11%). For the binary classification task, the model achieved an accuracy of 83.70%, sensitivity of 83.77%, and specificity of 83.75% while for the region segmentation task, an Intersection over Union (IoU) score of 0.648 for the AG regions and 0.777 for the incisura region. The results suggest that the developed model based on the UNet++ and Resnet50 architectures can effectively distinguish between AG and non-AG cases, and it can also be used to delineate specific regions of interest within the endoscopic images.

Based on a research carried out by [23], images of non-cancerous lesions and early gastric cancers (EGC) were used to evaluate a convolutional neural network’s diagnostic potential. A dataset, comprising of 386 non-cancerous lesions images and 1702 ECG images, was used for the training of the CNN model. The analysis results showed a sensitivity level of 91.18% showcasing the model’s adeptness to rightly identify EGC cases and a specificity level of 90.64% indicating its ability to properly identify non-cancerous lesions. Substantially, reaching an accuracy level of 90.91% of the CNN model to diagnose both types of cases. Upon comparison, no remarkable differences were found between the specificity and accuracy levels of the AI-aided system and endoscopy specialists. However, the specificity and accuracy levels of the non-experts were below those of both the endoscopists and AI-aided system. According to the study findings, the CNN model exhibited exceptional EGC and non-cancerous lesions diagnostic performance. Consequently, this research demonstrates the potential of AI-aided systems in assisting medical practitioners.

In the study presented by [24], an automated detection approach utilising CNN was proposed to assist in the identification of EGC in endoscopic images. The method employed transfer learning on two distinct classes of image datasets: cancerous and normal. These datasets provided detailed information regarding the texture characteristics of the lesions and were obtained from a relatively limited dataset. The CNN based network was trained using transfer learning techniques to leverage the knowledge acquired from pre trained models. By utilising this approach, the network achieved a notable accuracy of 87.6%. Subsequently, an external dataset was used for the evaluation of the model’s performance, an accuracy of 82.8% was attained. These results suggest that the proposed automated detection method based on CNN, trained on the cancerous and normal image datasets, effectively aids in the identification of EGC in endoscopic images. The achieved accuracy of 87.6% on the training dataset demonstrates the model’s ability to discern between cancerous and normal instances. Furthermore, the comparable accuracy of 82.8% on the external dataset indicates the model’s generalizability and potential for practical application in clinical settings.

The concept of SHAP for interpretable real time deep neural networks was introduced by [25]. The proposed technique showcased improved real time performance compared to existing methods. Experimental results highlighted the superiority of this approach over current deep learning techniques. Moreover, the author successfully addressed the needs of colorectal surgeons by providing satisfactory operational effectiveness and interpretable feedback. By incorporating SHAP, the technique not only offers enhanced performance but also ensures interpretability, aligning with the requirements of medical professionals in the field of colorectal surgery.

Upon investigation of prior research on the detection of gastrointestinal cancer using AI assistance, it became evident that this field will highly benefit from further exploration. While several AI models have been utilised to discover deformities in medical images, there remains a notable gap in the development of human comprehensible models that can provide explanations for model predictions. Although there has been a recent surge of interest among researchers, only a limited number of studies have focused on creating AI models that offer interpretability, allowing healthcare professionals and stakeholders to understand and trust the predictions made by these models. Therefore, there is a clear need for more research efforts to develop AI models in gastrointestinal cancer detection that not only achieve high accuracy but also provide comprehensible explanations for their predictions.

## 3 Materials and methods

**Fig 3:**
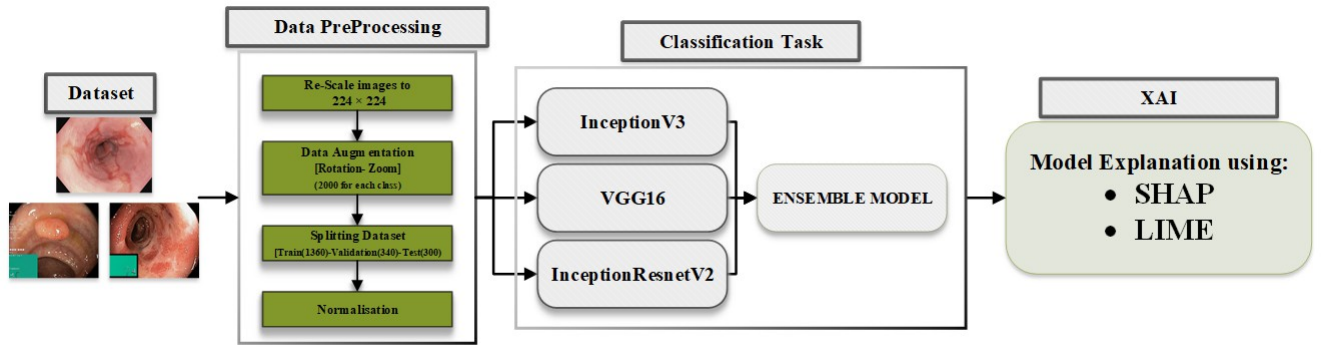
Structure of proposed model.

This section outlines the architecture of the proposed method, which incorporates XAI, and introduces the XAI based ensemble model for GI cancer detection. Fig 3 depicts the proposed structure of the XAI based GI Cancer detection system is presented. The system utilizes pathological findings obtained from the KvasirV2 dataset for training and evaluation purposes. To enhance the performance and accuracy of the system, an ensemble model is developed. This ensemble model combines the predictions of multiple models, potentially leveraging their complementary strengths and improving overall classification performance. Furthermore, an XAI technique is employed to uncover the deterministic features associated with each class. This technique allows for the interpretation and visualisation of the important features that influence the classification decisions made by the system. By integrating XAI into the ensemble model and analysing the deterministic features, the proposed method aims to provide insights into the decision making process of the GI cancer detection system, enhancing its transparency and interpretability.

### 3.1 Dataset

**Fig 4:**
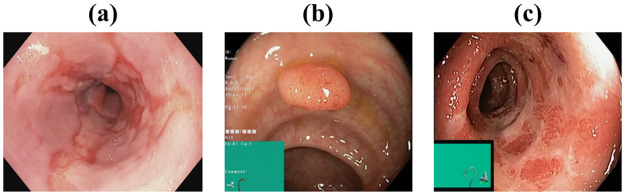
Example of pathological revelations [26] (a): Esophagitis, (b): Polyps, (c): Ulcerative Colitis.

Datasets play a crucial role in the advancement of various computing domains, particularly in the field of deep learning applications. The quality and availability of datasets are vital as they need to be appropriately labeled, exhibit diversity among images, and contain a sufficient number of instances. Several investigators and institutions have expanded the datasets for medical imaging so that it becomes easier to train and evaluate suggested models. This study make use of the Kvasir dataset, initially introduced by [26] in 2017, which composes of images that have been meticulously validated and annotated by medical experts. Each class contains 1000 images, thus showcasing pathological revelations, endoscopic approaches and anatomical landmarks within the gastrointestinal tract. The dataset can be accessed from [26]. However, for the purpose of this research, our focus was solely on the pathological findings class depicted on Fig 4, which encompasses three distinct categories:

1. **Esophagitis**: an inflammation induced mucosa break in the esophagus.
2. **Polyps**: structural abnormalities (lesions) in an organ ascertained as mucosal outgrows.
3. **Ulcerative Colitis**: a chronic condition causing inflammation of the colon and rectum.

To enhance the diversity and variety within the dataset, data augmentation techniques were applied to the original dataset. Specifically, rotation and zoom techniques were utilised to create variations of the existing images. This process involved rotating the images at different angles and applying zooming operations to produce new perspectives and scales. By applying these data augmentation techniques, another dataset having 2000 images per class was generated. This increased dataset size provided a broader range of image variations and ensured a more comprehensive representation of the pathological findings within the GI tract. The augmented dataset with its increased variety and enlarged sample size is crucial for training and evaluating the proposed models effectively. It enables the models to learn from a more diverse set of examples and improves their ability to generalize and make accurate predictions on unseen data. Our enhanced KvasirV2 dataset were separated into two parts: 85% (training + validation) and 15% testing.

### 3.2 Model development

The primary deep CNN that was implemented in the development of the ensemble model was InceptionV3, created by [27] in 2015. InceptionV3 is an upgraded version of the GoogleNet (Inception V1) and comprises of 42 layers. The second model utilised in developing the ensemble model was VGG16 which was established by [28] in 2014. It comprises of 16 layers and employs softmax as classifier. Finally, the InceptionResNetV2 was effectuated. InceptionResNetV2 is deep CNN having Inception Architecture as its foundational basis though it makes use of residual connections instead of undergoing the filter concatenation phase. It comprises of 164 layers and was developped in 2016 by [29]. Our enhanced models consisted of removing the classifier layer of the respective network and the addition of an average pooling, a flatten layer, a dense layer of 120 units with ReLu activation function and finally, a dense layer of 3 units with softmax. All three models were then finetuned by unfreezing and training some of the pre-trained layers along with the added layers.

**Fig 5:**
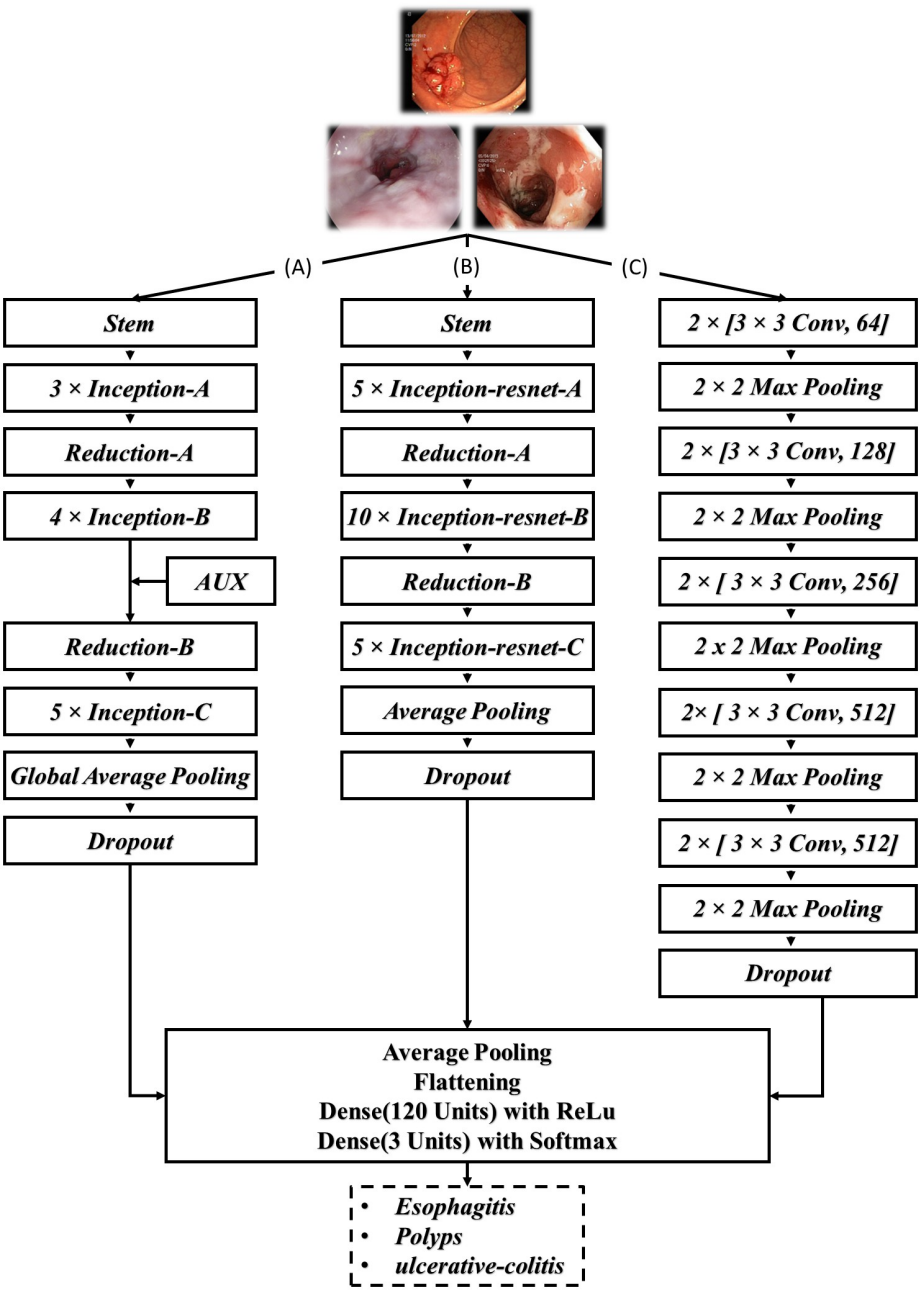
Model architecture. (A): InceptionV3; (B): InceptionResnetV2; (C): VGG16.

Fig 5 illustrates the architecture of the individual models.

#### 3.2.1 Ensemble model

Ensemble models are a valuable technique in machine learning that combines multiple individual models to enhance the overall performance of a system. The fundamental concept behind ensemble modeling is to leverage the strengths of different models to compensate for their respective weaknesses, resulting in improved accuracy, robustness, and generalisation capabilities. There are various types of ensemble models, including bagging, boosting, and stacking.

In bagging, multiple models are independently trained on different subsets of the training data. The final prediction is typically obtained by aggregating the predictions of all the models, using techniques such as averaging or majority voting. This approach helps to reduce overfitting and increase the stability of the predictions. Boosting, on the other hand, involves training models iteratively. Each new model focuses on the examples that were misclassified by the previous models, thereby progressively improving the overall performance. Boosting algorithms assign higher weights to difficult examples, allowing subsequent models to prioritize those instances during training. Stacking takes a different approach by utilising the predictions of multiple models as input features for a meta model. The meta model is trained to learn how to combine these predictions effectively and make the final prediction. This approach can capture complex relationships between the base models’ outputs and potentially improve overall performance.

Ensemble models find applications in various domains of AI, including computer vision, natural language processing, and speech recognition. For instance, in image classification tasks, an ensemble of CNN can be employed to enhance accuracy and robustness. Each CNN within the ensemble may specialize in different aspects of feature extraction or classification, leading to improved classification performance. Ensemble models are a powerful technique in machine learning that leverages the collective wisdom of multiple models. By combining diverse models, ensemble methods can mitigate individual model limitations and yield superior performance across a range of AI applications [30–32].

**Fig 6:**
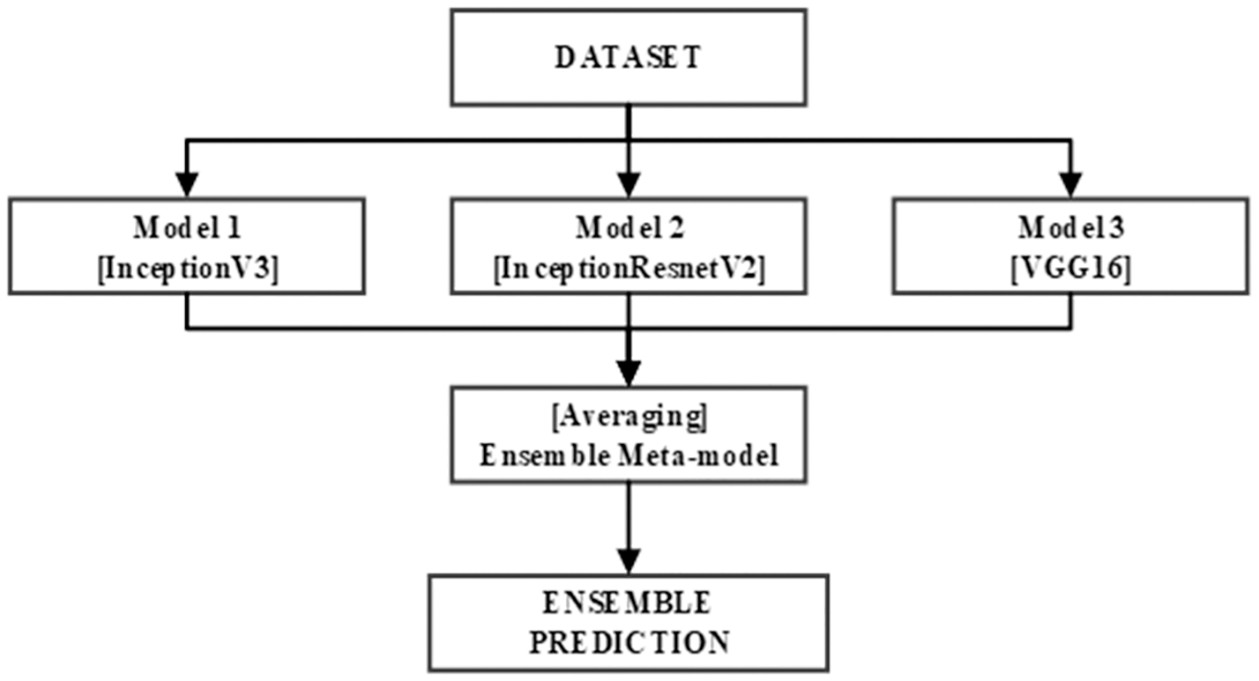
Architecture of the ensemble model.

This study focuses on the development of an ensemble model based on bagging technique. This is executed through the synthesis of the predictions of three pre-trained CNN : InceptionV3, InceptionResNetV2 and VGG16. Fig 6 illustrates the architecture of the ensemble model.

Moreover, each of the three previously mentioned CNN models was applied on our enhanced KvasirV2 dataset which was separated into two parts:85% (training + validation) and 15% testing. After individual training of the models, the average approach was used to develop the ensemble model through the combination of each model’s predictions. The average technique formulates an average of the predictions obtained from the three trained models resulting into the generation of the final prediction.

### 3.3 XAI in medical imaging

The field of XAI is experiencing rapid growth, focusing on enhancing transparency and interpretability in machine learning algorithms. This advancement is of particular significance in the realm of medical imaging, as the outputs of machine learning models greatly influence patient care. XAI methods play a crucial role in enabling clinicians and radiologists to comprehend the rationale behind the model’s predictions, thereby instilling confidence in the accuracy of the model’s assessments. Moreover, XAI techniques aid in the identification of potential biases within the model, facilitating the prevention of misdiagnosis and promoting equitable healthcare outcomes [11, 12, 33].

XAI in medicine and healthcare have been classified in five categories by [13]. The purpose of our research is to make medical imaging more explainable which have led to us exploring the XAI technique of explanation through feature relevance, SHAP and LIME are examples of such method.

#### 3.3.1 Shapley additive explanations

SHAP method developed by [14] is a model agnostic technique derived from cooperative game theory, enabling the interpretation of machine learning model outputs by quantifying the contribution of each feature. It provides a comprehensive framework that considers both global and local feature importance, accounting for feature interactions and ensuring fairness in assigning importance. The SHAP values align with desired axioms of feature attribution methods, including local accuracy, consistency, and missingness. Local accuracy ensures that the sum of SHAP values corresponds to the discrepancy between the model’s prediction and the expected output for a specific input. Consistency guarantees that fixing a feature’s value will not decrease its associated SHAP value. Missingness implies that irrelevant features have SHAP values close to zero.

#### 3.3.2 Local interpretable model-agnostic explanations

LIME introduced by [15], is a widely used technique for interpreting AI models. Its approach involves generating understandable explanations tailored to individual predictions made by the model. LIME works by generating local, interpretable explanations for individual predictions made by a model. It does this by perturbing the image and observing how the model’s predictions change. By fitting a simple, interpretable model to these perturbed data points in the vicinity of the original instance, LIME approximates the behavior of the complex model in the local neighborhood of interest. In image classification, LIME can highlight specific areas of an image that contribute most to the model’s decision for a particular class, providing insights into the model’s decision-making process on a per-instance basis.

Various applications have benefited from SHAP values and LIME, encompassing domains such as image recognition, natural language processing, and healthcare. For instance, in a study focusing on breast cancer detection, SHAP values were utilised to identify the most relevant regions in the images [34]. Similarly, in another study concerning the detection of relevant regions in retinal images for predicting disease severity [35], SHAP values were employed to interpret the features of a deep neural network model. [36] used the LIME approach to explain the classification outcome for a Skin cancer classification model. Likewise, [37] applied LIME to visualise prostate cancer.

## 4 Result and discussion

### 4.1 Model implementation

The initial phase of the experimental methodology involved the development of an ensemble model tailored for the classification of gastrointestinal lesions. This phase entailed the independent training of three pre-trained CNN, specifically the InceptionV3, InceptionResNetV2, and VGG16 models, using the Kvasir dataset. Subsequently, these individual models were amalgamated to construct the ensemble meta-model. To adapt the underlying architectures of the aforementioned pre-trained CNNs, a global average pooling layer was integrated into the model architecture. This pooling layer served the purpose of synthesizing spatial information from preceding layers while simultaneously reducing the dimensionality of feature maps. Following the integration of the pooling layer, a dropout layer with a dropout rate of 0.3 was incorporated into the model architecture to address concerns related to overfitting, thereby enhancing the generalisation capabilities of the model. Finally, the Softmax activation function was employed for classification, facilitating the assignment of probabilities to each class and enabling predictions pertaining to GI lesion classes by both individual models and the ensemble model. The codes are available at https://github.com/mmuzzammil-auzine/XAI-based-Ensemble-model-for-Gastrointestinal-pathologies.git.

### 4.2 Training setup

The experimental setup involved the training of both individual models and the ensemble model using K-fold cross-validation, with K set to 5. Each fold underwent 10 epochs of training, with a fixed batch size of 32. The Adam optimisation algorithm was used for model optimisation, leveraging sparse categorical cross-entropy as the loss function. This amalgamation of optimisation strategies facilitated the effective training of both individual models and the ensemble model. The utilisation of the Adam optimisation algorithm ensured the adaptability of the model weights based on the calculated gradients, thereby enhancing performance and accuracy in the classification of GI lesions. This experimental configuration was meticulously devised to ensure robustness and reliability in evaluating the efficacy of the proposed ensemble model for GI lesion classification.

#### 4.2.1 Hardware specification

Our models were trained on a high-performance laptop equipped with an 11th Gen Intel^®^ Core™ i7-11800H processor, 32GB of RAM, and an NVIDIA RTX A3000 Laptop GPU with 6 GB of dedicated memory. This machine offered us substantial computing power and memory capacity to effectively trained our models and enabled us to achieve a robust performance in our classification tasks.

### 4.3 Experimental results

**Fig 7:**
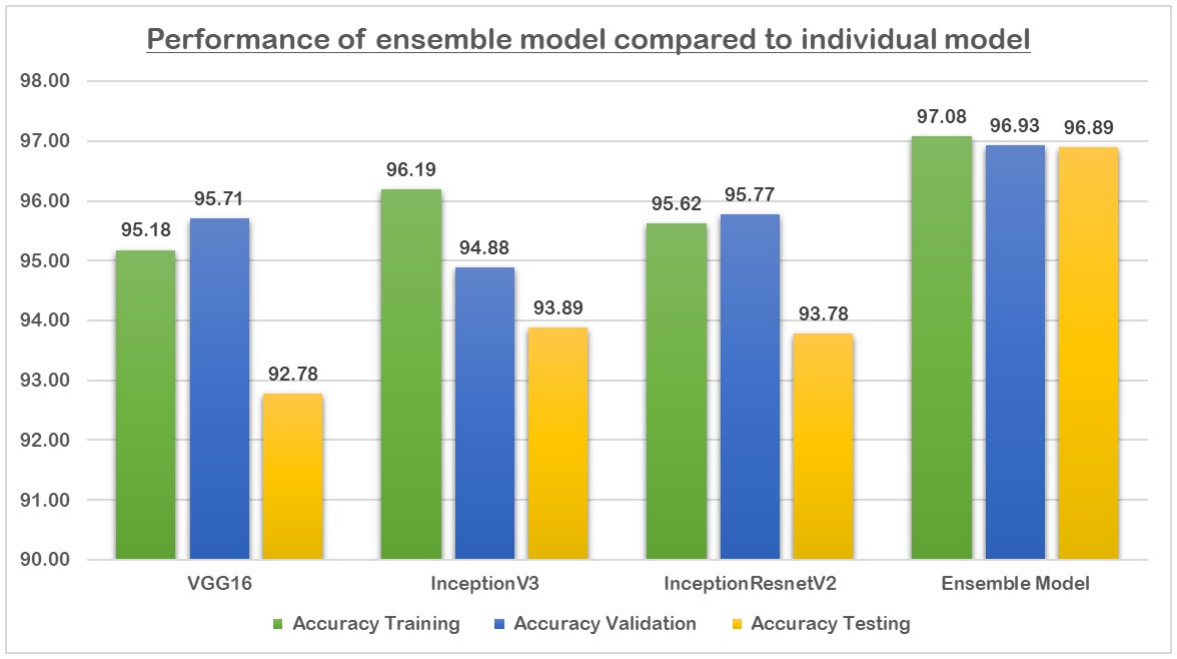
Ensemble model compared to the individual models.

By leveraging the strengths of multiple pre-trained CNN models through ensemble learning, the developed model aimed to enhance the accuracy and robustness of GI lesion classification. Fig 7 shows the classification results attained during the separate training of the CNNs models along with the developed ensemble model.

Analysing the results illustrated in Fig 7, we can observe that the ensemble model outperforms all individual models on all datasets. The ensemble model shows consistent high accuracy across all datasets, with minimal drop in performance from training/validation to testing. This suggests that the ensemble model generalizes well to unseen data. Each individual model demonstrates strong performance on both training and validation datasets. However, there is a slight drop in accuracy when tested on unseen data, indicating some level of overfitting. The differences in architectures (VGG16, InceptionV3, InceptionResNetV2) contribute to variations in performance, but overall, they perform similarly. While the individual models exhibit some degree of overfitting, they serve as valuable components in the developement of the ensemble model, which addresses these weaknesses and yields superior performance.

**Fig 8:**
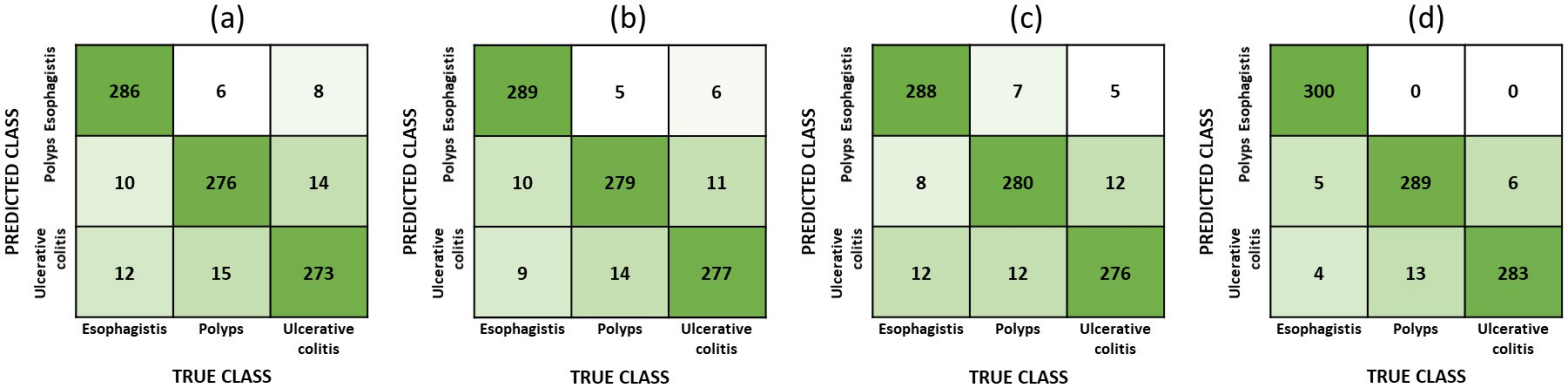
Confusion matrix [(a): VGG16, (b): InceptionV3, (c): InceptionResnetV2, (d): Ensemble model].

Based on Fig 8, the averaged F1-score, recall, and precision for each model regarding each class (esophagitis, polyps, and ulcerative colitis) are presented in the classification report, depicted in Table 1.

**Table 1:** Models’ classification report.

Model	Accuracy(%)	Average		
		Precision(%)	Recall(%)	F1-Score(%)
VGG16	92.778	92.778	92.776	92.769
InceptionV3	93.889	93.889	93.891	93.881
InceptionResnetV2	93.778	93.778	93.783	93.771
Ensemble Model	96.889	96.889	96.902	96.877

Fig 8 displays the confusion matrix obtained from the classification results. The confusion matrix provides an overview of the model performance by showing the number of correctly and incorrectly classified samples for each class. The visualisation of the confusion maps from Fig 8 indicates that the ensemble model has significantly enhanced the classification task, as it only misclassified 28 samples, compared to 65, 55 and 56 misclassified samples from VGG16, InceptionV3 and InceptionResnetV2 respectively.

From Fig 8 we can also observe that the ensemble model has achieved perfect precision on the esophagistis class, that is, the model was able to classify all samples of esophagitis correctly. The ensemble model only misclassified 11 polyps samples and 17 ulcerative colitis samples.

Table 1 displays the classification report, which includes the average F1-score, recall, and precision metrics for each model based on each class: esophagitis, polyps, and ulcerative colitis. The F1-score is a measure of the model accuracy, combining precision and recall into a unified metric. Recall represents the model ability to correctly identify positive samples, while precision reflects the model ability to correctly classify positive predictions. These metrics provide insights into the model performance for each specific class. Both the confusion matrix and the classification report offer valuable information to evaluate the accuracy and effectiveness of the developed ensemble model in classifying gastrointestinal lesions.

The results obtained from the ensemble model demonstrate a significant improvement in the overall accuracy compared to the individual models. The classification report also showcases high precision, recall, and F1-score for across all three classes: esophagitis, polyps, and ulcerative colitis. These metrics indicate that the model is capable of accurately identifying positive instances and minimising false positives and false negatives. With an overall F1-score of 96.877% and an overall accuracy of 96.89%, the ensemble model exhibits strong performance in classifying gastrointestinal lesions. The high F1-score also suggests that the model achieves a balance between precision and recall, indicating its capability to correctly identify both positive and negative instances.

Given the importance of accurate prediction in the context of gastrointestinal cancers, the promising results of the ensemble model indicate its potential for further development and application in clinical settings. The high F1-scores and overall accuracy provide evidence of the model’s effectiveness in aiding GI cancers diagnosis, making it a valuable tool in healthcare practice.

### 4.4 Model explanation using SHAP and LIME

To gain comprehensive insights into the deterministic features contributing to the predictions of our ensemble model, we employed both the SHAP partition explainer with a blurring-based masker and the LIME technique. This combined approach allowed us to delve deeper into understanding the inner workings of our model and provided a more nuanced explanation for its predictions.

Utilising SHAP in conjunction with LIME, we were able to visualize specific areas of the image that played a crucial role in the model predictions. This comprehensive analysis not only highlighted deterministic features but also provided a more holistic understanding of the model’s decision-making process.

For this analysis, we have used images from each class (esophagitis, polyps, and ulcerative colitis) from the test set. By applying both SHAP and LIME techniques, we obtained visual representations and explanations for the contributing characteristics for each class. This dual-method approach enhanced the interpretability of our model’s predictions and provided valuable insights into its decision-making mechanisms.

**Fig 9:**
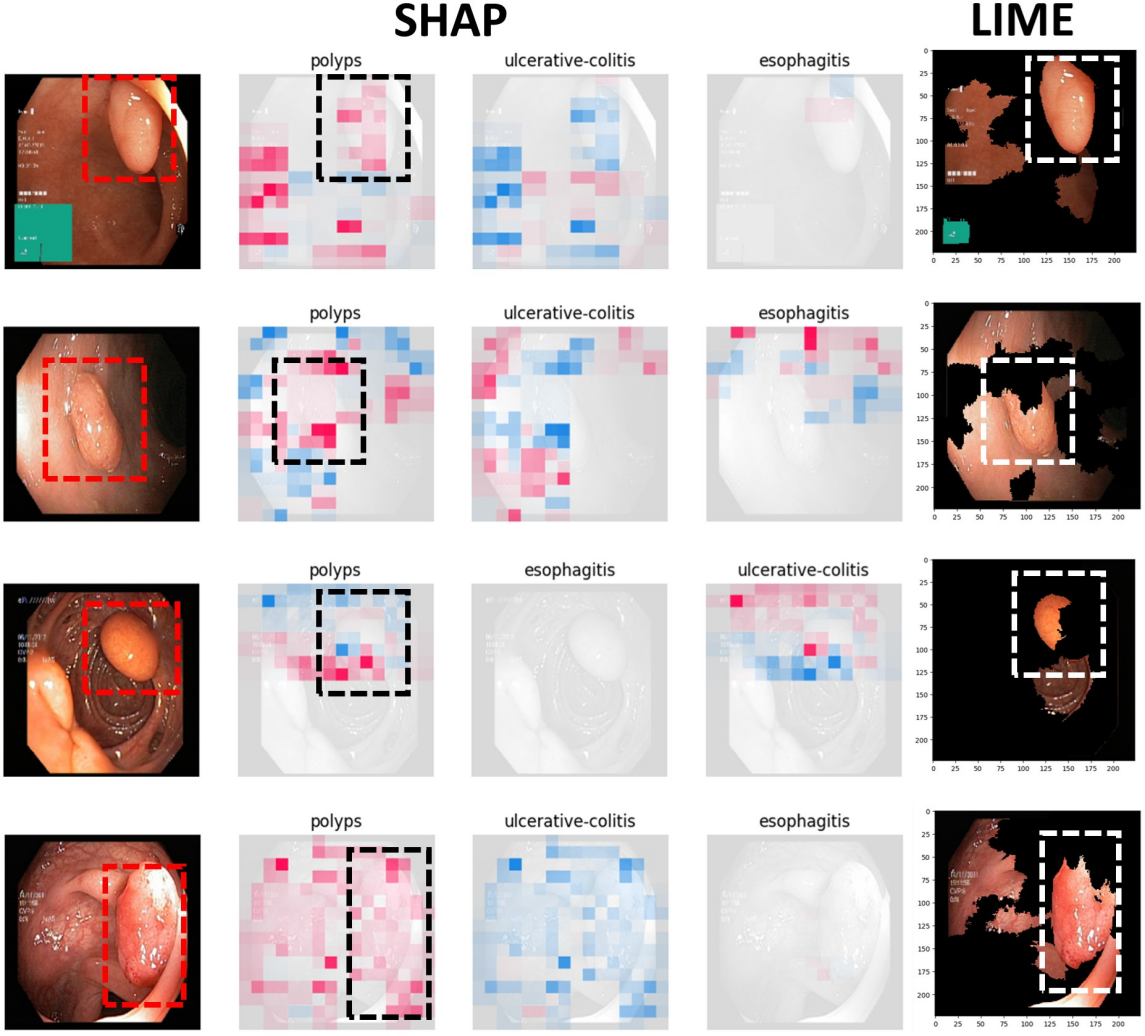
Correctly classified polyps.

**Fig 10:**
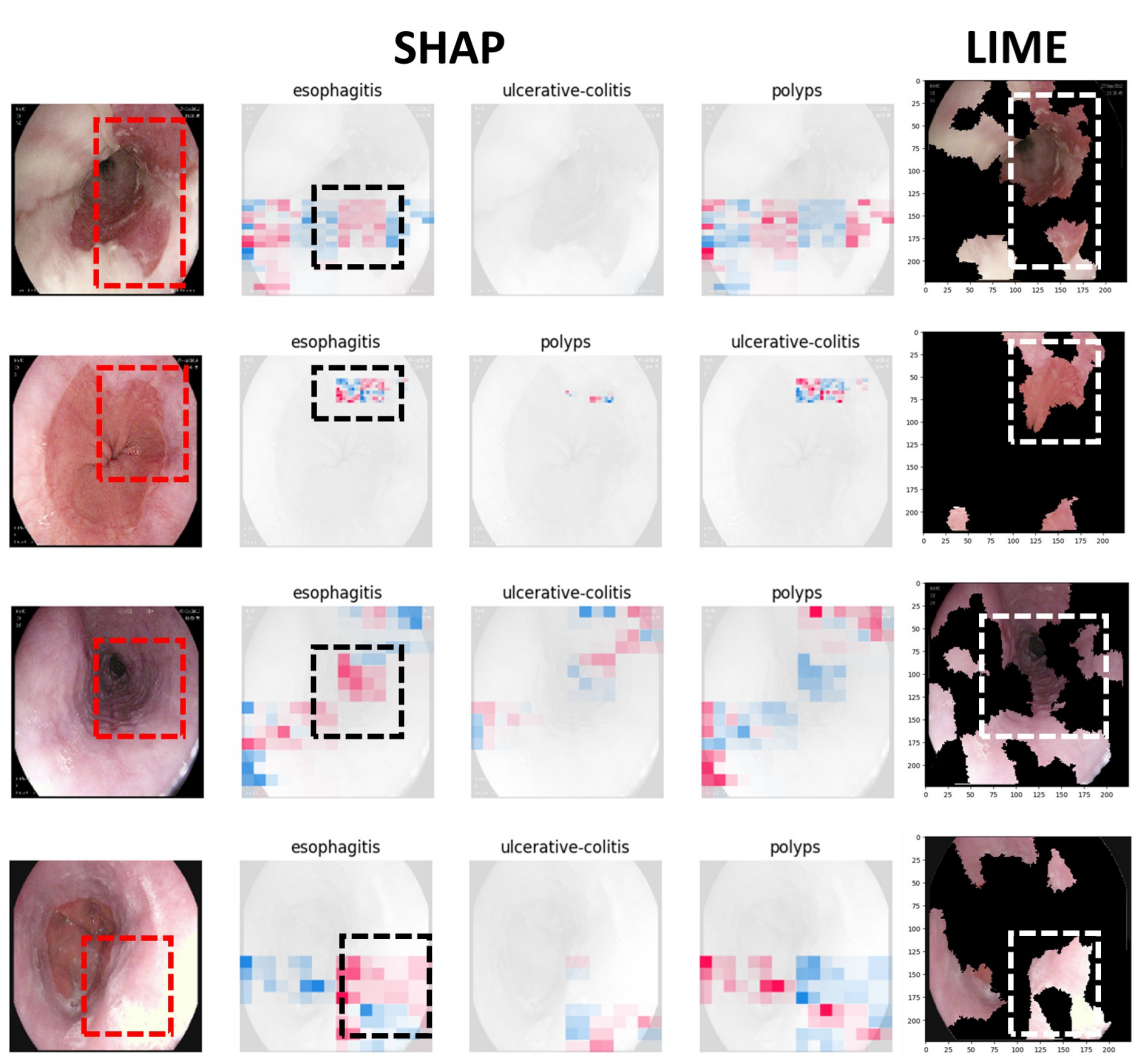
Correctly classified esophagistis.

**Fig 11:**
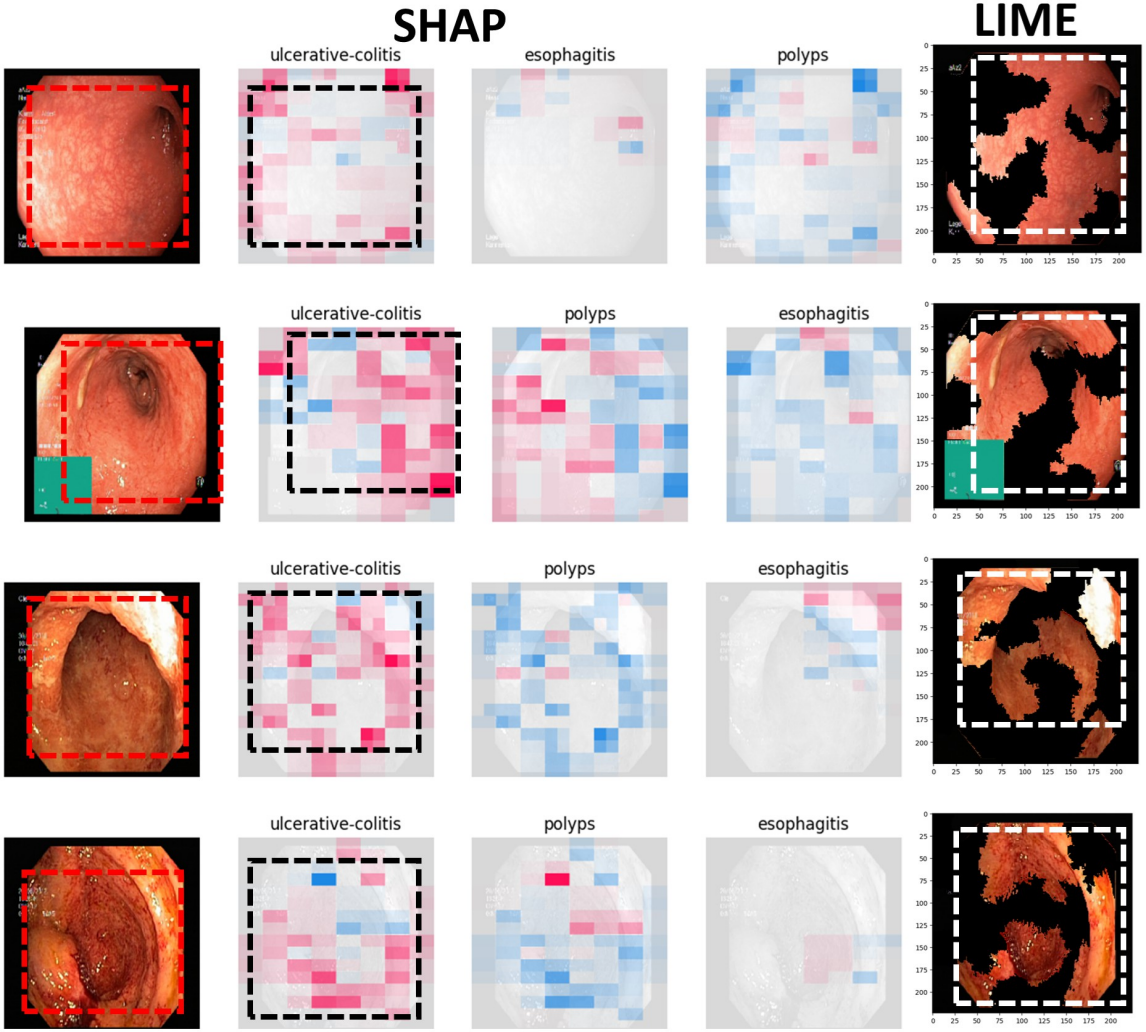
Correctly classified ulcerative colitis.

**Fig 12:**
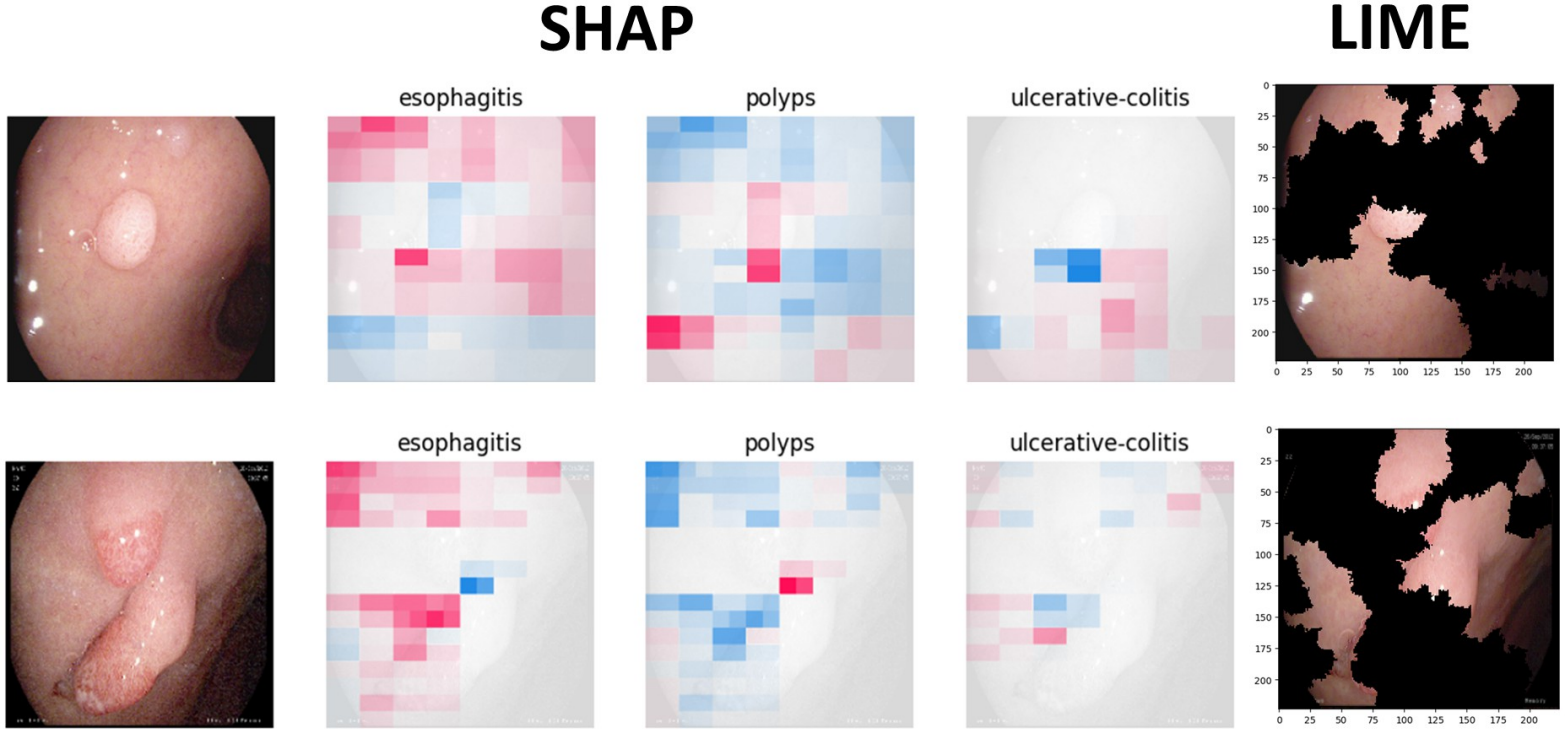
Polyps classified as esophagistis.

**Fig 13:**
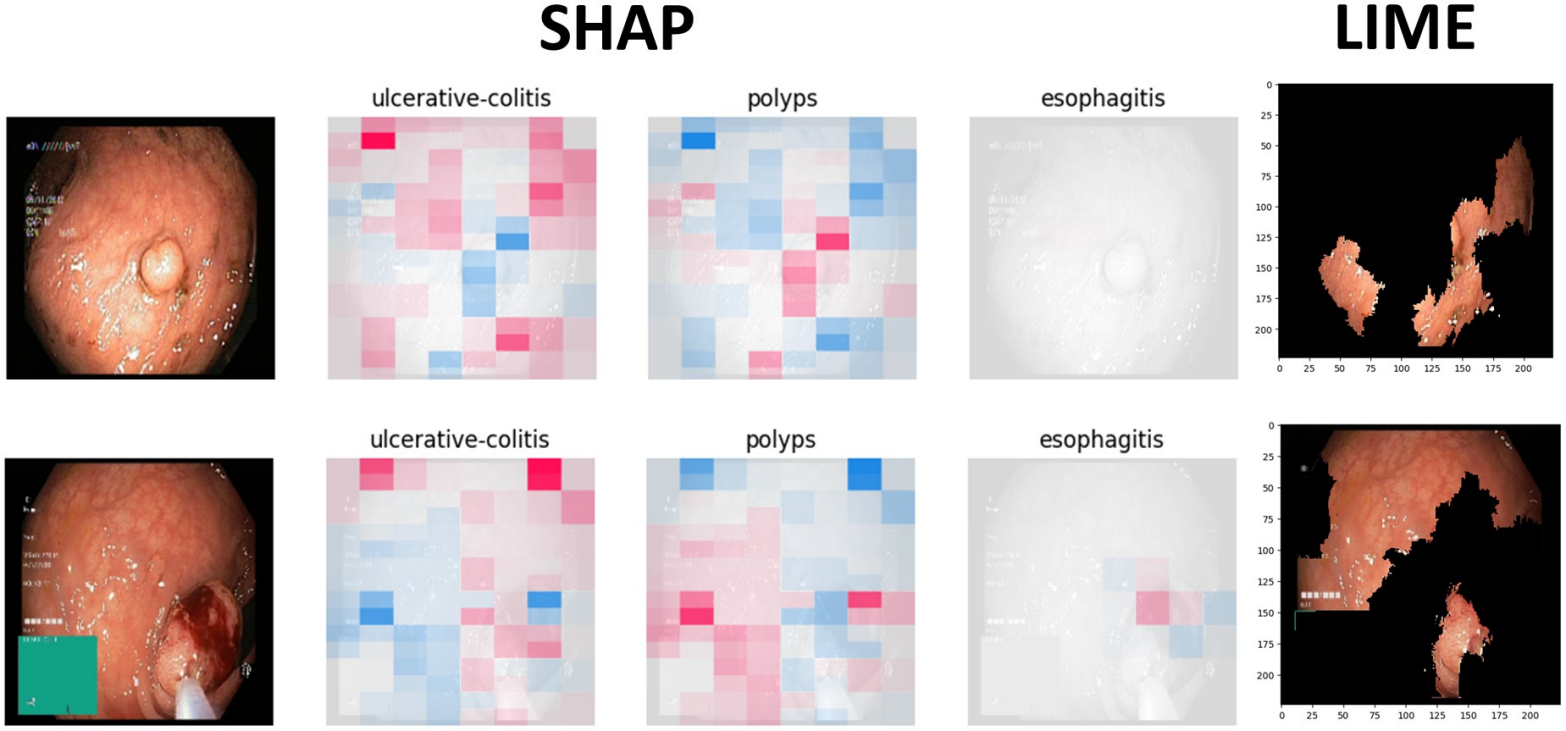
Polyps classified as ulcerative colitis.

**Fig 14:**
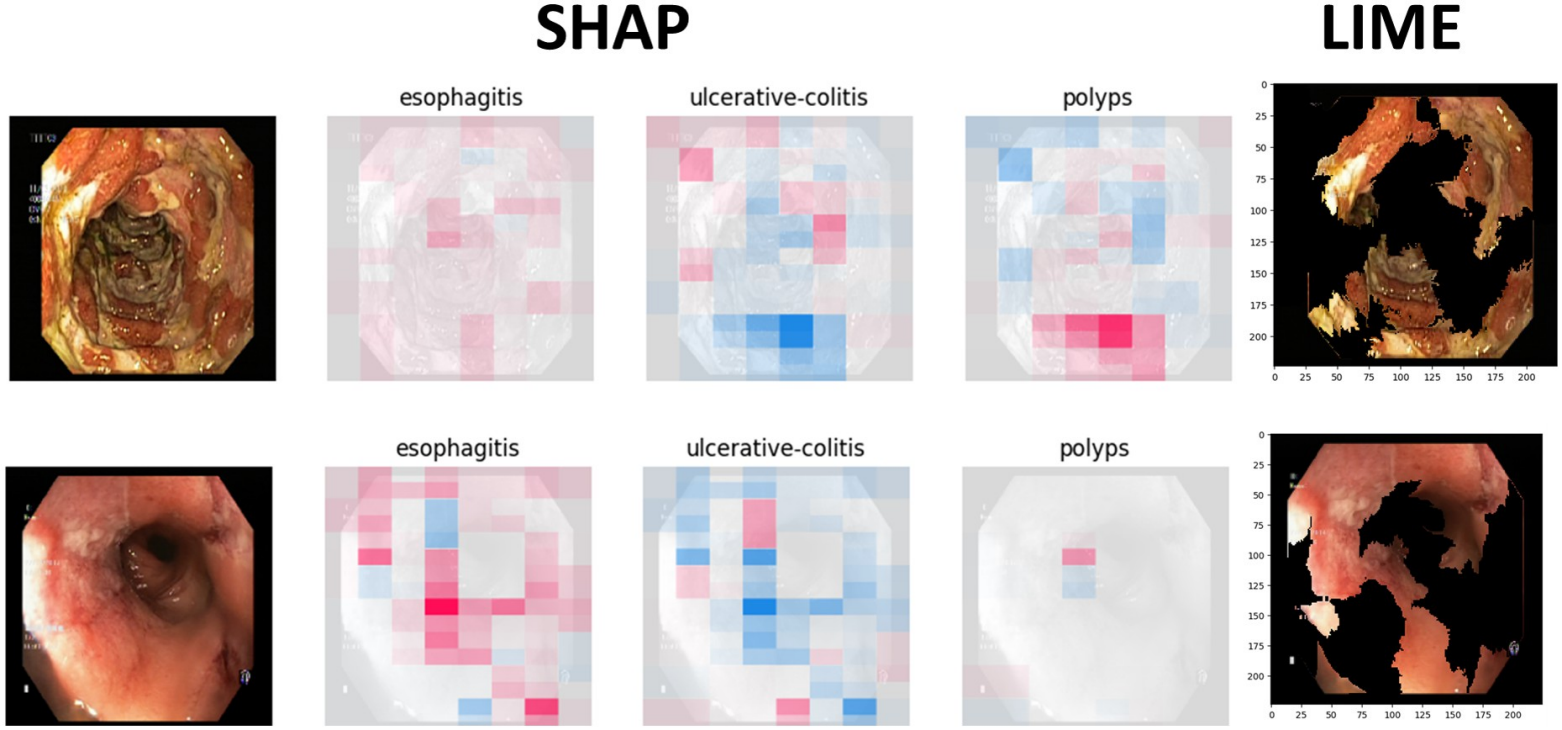
Ulcerative colitis classified as esophagitis.

**Fig 15:**
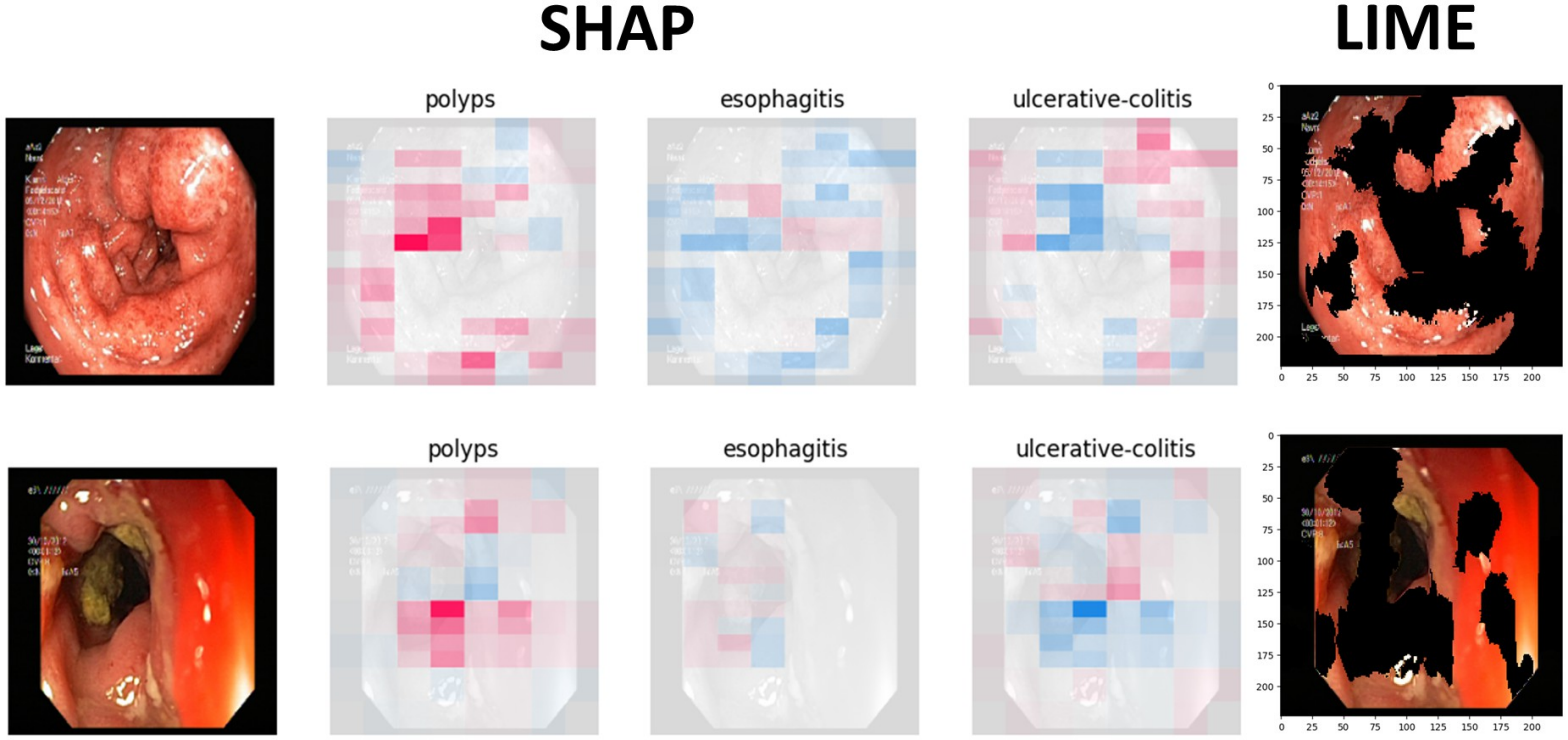
Ulcerative colitis classified as polyps.

Figs 9 to 11 illustrate the deterministic features and their importance for the correctly classified esophagitis, polyps, and ulcerative colitis classes. On the other hand, Figs 12 to 15 illustrate the misclassified classes. These visualisations provide valuable insights into the specific regions or patterns within the images that significantly influenced the ensemble model’s decision making process, thus enhancing the interpretability and explainability of our model’s predictions.

The XAIs’ results illustrated from Figs 9 to 15 consists of 3 sections. The first one is the actual test image, the next section consist of three images for the SHAP results and finally, the LIME result.

The SHAP results are based on SHAP values, which are illustrated on the chart visually representing the actual image with specific sections emphasised in both blue and red hues. In addition to highlighting elements contributing to the prediction, the SHAP chart also indicates the class to which the image was predicted. The first image corresponds to the class predicted by the model as the most likely category, while the subsequent two images represent the next two classes in order of likelihood. In this context, the red color highlights elements within the image that positively contributed to the prediction of the primary class, while the blue color denotes areas that had an adverse impact on the prediction. These visual cues offer insights into the decision-making process of the model and the relative importance of different image regions in determining the final prediction for each class. This visual interpretation aids in understanding which aspects of the image are driving the model’s predictions and facilitates the identification of relevant features for further analysis and validation.

For the LIME results, our model displays the first five most contributing features to the prediction. Following this, the model segments the remaining features and highlights the subsequent five most influential features. This approach allows for a focused examination of the key elements driving the model’s predictions. By prioritising the most significant features initially, we gain immediate insights into the primary factors shaping the model’s decision-making process. Subsequently, the segmentation of additional features provides a more comprehensive understanding of the various contributing factors, allowing for a deeper analysis of the image’s predictive characteristics. This structured approach to feature selection and segmentation enhances the interpretability of the LIME model’s predictions, enabling us to pinpoint specific image attributes that significantly influence the model’s output. Such insights are invaluable for refining the model and validating its predictive capabilities.

#### 4.4.1 Correctly classified samples

Figs 9 to 11 illustrates the results of the correctly classified samples for polyps, esophagitis and ulcerateive colitis respectively. Taking the first image from Fig 9 which showcases the results of the polyps sample as a reference, the pathology, which in this case is polyps, is bounded by the red box. From the SHAP chart, we can deduce that the first class predicted is ‘Polyps’, indicating that the model correctly classified the sample into its correct class. Moreover, the area bounded by the black box encompasses the section containing the polyps. We observe predominantly red hues within this area, indicating features that positively contributed to the prediction, as previously detailed. Furthermore, upon examining the subsequent two classes predicted by the model, we notice that both images predominantly exhibit blue hues or missingness in the area associated with the polyp pathology. This suggests that these regions negatively influenced the model’s prediction for these classes. Regarding the LIME results, we note that the section consisting of the polyps, bounded by the white box, is among the top five features, as detailed above. This emphasizes the significance of this region in contributing to the model’s prediction. The same approaches was followed for the subsequent two classes.

Overall, the model predicts and outputs the deterministic features of each tested image, highlighting the regions that contribute positively or adversely to the predicted categories. This provides valuable insights into the specific image characteristics that the model considers when making its predictions.

### 4.5 Misclassified samples

Figs 12 to 15 illustrate the results of the misclassified samples for polyps and ulcerative colitis. As mentioned in the previous section, our ensemble model achieved perfect precision for the esophagitis class, resulting in zero misclassified samples for esophagitis. The figures below illustrate misclassified samples in their respective combinations.

From Fig 12, we can observe the misclassification of polyps sample as esophagistis. One plausible explanation for these misclassifications could be attributed to the similarity in the features (such as the lining) of the esophagistis.

In Fig 13, the samples clearly show polyps. The model might have been confused by the presence of blood, leading it to incorrectly classify them as ulcerative colitis. It’s important to note that both polyps and ulcerative colitis can show a loss of vascularity. However, polyps are characterised by mucosal outgrowths, which help distinguish them from ulcerative colitis.

In Fig 14, these samples exhibit tubular structures with ulcers in the colonic mucosa. However, it is conceivable that the model misinterpreted these samples due to their similarity with features typically associated with both colonic ulcers (as seen in ulcerative colitis) and esophageal ulcers (as in esophagitis).

In Fig 15, one significant factor contributing to this misclassification could be attributed to the distended structure of these colon samples. The distension might have led to confusion within the model, as it shares resemblances with the features of polyps, characterised by mucosal outgrowths.

Differentiating between polyps and ulcerative colitis relies on recognising specific features. Both conditions often display a loss of vascularity and erythema. However, it’s crucial to note their physical characteristics: polyps typically grow outward, while ulcerative colitis tends to cause flat changes in the mucosal lining. Teaching the model to discern these differences during its training can improve its ability to accurately classify these conditions based on their distinct appearances.

### 4.6 Discussion

The limited number of studies conducted on gastrointestinal cancer detection highlights the need for further research in this area. Existing studies have reported moderate to high accuracies using deep learning models such as InceptionResNetV2 and InceptionV3. For instance, one study [38] achieved an accuracy of 84.5% using InceptionResNetV2 with a dataset of 854 images, while another study [23] reported an accuracy of 90.1% using InceptionV3 with a test set of 341 endoscopic images. In comparison, our optimised ensemble model, along with the individual models, demonstrates superior performance compared to these existing studies. The accuracy of our ensemble model is reported as 96.89% with an average F1-score of 96.877%. This indicates the effectiveness of our approach in accurately classifying gastrointestinal lesions. Table 2 summarises a comparison of our proposed model with existing works.

**Table 2:** Summary of comparison of proposed model with existing works.

Existing Work	Method	Accuracy %	Discussion
[22]	Unet++ & ResNet50	83.7	A binary class classifcation between gastritis and non gastritis was conducted. The work has not explored multiclass classification which is more challenging and also there was class imbalance in their datasets.
[23]	InceptionV3	90.1	CNN was used to classify gastric cancer lesions and non cancerous lesions. The authors achieved an accuracy of 90.1%. However, it was only for a binary classification and also there was class imbalance in their datasets.
[24]	CNN	87.6	CNN was used for a binary class classification. The work has not explored multiclass classification which is more challenging.
[38]	Inception ResnetV2	84.5	The authors conducted a binary class classication with Gastric ulcers. Multiclass classification was not investigated
[39]	ResNet-152 combined with Grad–CAM	93.46	The authors conducted a multi class classification using the Kvasir dataset and apply several versions of Grad Cam for explainability.
[40]	Xception with Grad–CAM	88.74	Using the HyperKvasir dataset, the authors conducted a multi class classification using the the Xception model with Contrastive then cost-sensitive learning. For XAI, they have applied CAM techniques.
Proposed Work	**Ensemble Model with XAI**	96.89	In our work, we have been able to classify Esophagitis,Polyps and Ulcerative Colitis with an overall accuracy of 96.89% and also provide a visual explanation of the deterministic features of each class.
	YOLOv8	97.3	We have conducted preliminary experiments using YOLO, we observed that it has achieved a satisfactory classification results. We will explore the potential of using YOLO as a future work

However, it is important to acknowledge the challenges faced in developing and evaluating deep learning models for gastrointestinal cancer due to the limited availability of publicly accessible datasets in this domain. This scarcity hinders the progress and thorough evaluation of deep learning models for gastrointestinal cancer detection.

Moreover, the lack of explainability in deep learning models has contributed to the hesitation among healthcare professionals in adopting these models in clinical practices. To address this limitation, our proposed model incorporates the SHAP technique, which allows for the identification of deterministic features within the images associated with gastrointestinal pathologies. By providing explanations for the model decision making process, our model enhances the interpretability and trustworthiness of the results.

Upon analysing the test images using both SHAP and LIME techniques, we observed complementary insights into the model’s predictions. SHAP provided a detailed breakdown of the specific features within the images that influenced the model’s decision, offering a comprehensive understanding of the deterministic factors contributing to each prediction. In the same essence, LIME provided localised explanations by highlighting the most relevant regions within the images that influenced the model’s output. This localised perspective offered additional granularity, allowing us to pinpoint specific areas of importance within the images. By combining the results from both SHAP and LIME, we obtained a rich and multifaceted interpretation of the model’s predictions, enhancing our confidence in its decision-making process and providing valuable insights for further refinement and interpretation.

The amalgamation of ensemble models and SHAP & LIME technique in our proposed model has shown great progress in the field of explainable AI in GI cancer diagnosis. By combining the strengths of multiple individual CNN models through ensemble learning, it further escalates the accuracy and robustness of the our model predictions. The SHAP & LIME methods also allow us to gain insights into the features that operate the model’s prediction. This elucidated aspect plays a salient and pivotal role in medical applications as it helps healthcare professionals to have a better understanding behind the model’s decisions. By pointing out the specific features that contribute to the prediction, we can enhance the trust, transparency and adaptation of the the model in a clinical settings. The high level of accuracy achieved by our proposed model, with its ability to showcase the deterministic features, makes it a potential tool for improving gastrointestinal cancer diagnosis. It has the potential to assist medical professionals in making more detailed decisions and improving patient outcomes.

However, it is important to note that further research, validation, and collaboration with healthcare experts are necessary to refine and optimize the model for real world clinical applications. By continuing to advance XAI models in the field of gastrointestinal cancer diagnosis, we can unlock new possibilities for improved patient care and outcomes.

## 5 Conclusion

The implementation of AI technology in the medical field has mostly been challenged due to the lack of explainability. This research addressed this problem by delving into the SHAP & LIME technique, SHAP & LIME makes the extraction of predetermined characteristics from pathological results of GI cancers possible. We introduced SHAP & LIME in our study with the objective to boost the explainability and interpretability of our model’s predictions. Our research begins with the development and training of an enhanced ensemble model. Three pre trained CNN models: InceptionV3, InceptionResNetV2, and VGG16 were combined using the averaging technique. These models were trained on the pathological findings of the KvasirV2 dataset, which is a valuable resource in the field of gastrointestinal cancer diagnosis. The strengths of these models were maximised through ensemble learning with the aim to enhance the effectiveness and accuracy of our model. The diagnosis of cancer through the ensemble model can become more robust and reliable as it combines the individual strengths and abilities of each component model. Furthermore, the SHAP & LIME explainer algorithm were employed to reveal the relevant features associated with each pathology. This technique allows us to understand which specific features and regions of the medical images contribute to the model’s predictions. By extracting and visualising these features, we can gain valuable insights into the decision making process of the model and improve our understanding of the underlying factors influencing the predictions. The findings of our research shows that the evolution of explainable AI models for cancer diagnosis, particularly in the field of gastrointestinal cancers, is progressing in an optimistic and favorable manner.
